# Phytochemistry and Pharmacological Activities of *Dracaena cinnabari* Resin

**DOI:** 10.1155/2021/8561696

**Published:** 2021-07-22

**Authors:** Yahya S. Al-Awthan, Omar Salem Bahattab

**Affiliations:** ^1^Department of Biology, Faculty of Science, University of Tabuk, Tabuk, Saudi Arabia; ^2^Department of Biology, Faculty of Science, Ibb University, Ibb, Yemen

## Abstract

*Dracaena cinnabari* (*D. cinnabari*) is an endemic plant located in Socotra Island, Yemen. Deep red resin attained from different plant species including *D. cinnabari* is commonly known as dragon's blood. In folk medicine, it is prescribed for the treatment of traumatic dermal, dental, and eye injuries as well as blood stasis, pain, and gastrointestinal diseases in humans. Numerous studies have investigated that this resinous medicine has antidiarrheal, antiulcer, antimicrobial, antiviral, antitumor, anti-inflammatory, analgesic, wound healing, and antioxidant activity. Several phytochemicals have been isolated from *D. cinnabari*, including the biflavonoid cinnabarone, triflavonoids, metacyclophanes, chalcones, chalcanes, dihydrochalcones, sterols, and terpenoids. The present review highlights the structures and bioactivities of main phytochemicals isolated from *D. cinnabari* regarding the botany and pharmacological effects of the resin derived from this plant.

## 1. *Dracaena cinnabari*: An Overview

### 1.1. Genus *Dracaena*

Genus *Dracaena* belongs to the family *Agavaceae* and contains xeromorphic species distributed in the Macaronesian islands, Madagascar, and along the African coast from Southern Africa into Arabia. Species of *Dracaena* include small much-branched trees or shrubs that are mostly deciduous and generally thorny. This genus is comprised of about 100 species in tropical and subtropical areas and produces a red resin from the sap [1]. The chloroplast genome of some species has recently been shown to be a “barcode” for *Dracaena* sp. identification [2].

### 1.2. *Dracaena* Resin: Dragon's Blood

It is a deep red resinous exudate that is acquired from cut stems of several species of genera *Pterocarpus*, *Dracaena*, *Croton*, and *Daemonorops* [3]. Six *Dracaena* plants growing in the Arabian Peninsula, Southeast Asia, and West Africa are main sources of this resin [4]. The resin is a commercially important export, especially from Socotra Island, Yemen, where it is known locally as Dam Alakhwin [5], and has been used in traditional medicine for the treatment of wounds, fractures, ulcers, dysentery, tumors, diarrhea, and diabetes [1, 2]. Recently, this resin was documented to have antioxidant and anti-inflammatory activity that promotes and enhances skin repair, blood circulation, immune function, and hemostasis [1, 6]. All of these effects are thought to be a result of the presence of many phenolic compounds, such as flavonoids, saponins, and terpenes, present in the resin [1]. In addition to its pharmaceutical uses, this resin has also been introduced as an art pigment by many cultures [2].

### 1.3. Plant Distribution

*Dracaena cinnabari* (*D. cinnabari*) is endemic to Socotra Island [7] and has been listed as a vulnerable species in Yemen according to the International Union for Conservation of Nature Red List [8]. This plant is found mostly in the highlands and mountains of central and eastern parts of the island at altitudes from 323 to 1483 m above sea level and missing from seaside plains and lowlands below 180 m above sea level [9]. In Yemen, *D. cinnabari* occupies only 5% of its current potential habitat according to Attorre et al. [10].

### 1.4. Botanical Description

**Figure 1:**
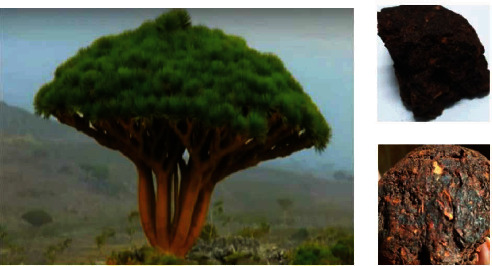
Photos of *D. cinnabari* (a) and its resin collected for marketing purposes (b).

*D. cinnabari* is usually 30–60 feet tall and has a straight or branched, strong trunk that is about 30 cm in diameter [11, 12]. Leaves are only found at the end of the youngest branches and are shed every 3 or 4 years before new leaves simultaneously mature. A distinctive growth habit is associated with *Dracaena* species that is known as “*dracoid habitus*” [13]. The flowers are found in clusters of 2–5 and carried on racemes or panicles. Its fruits are small fleshy berries containing 1–4 seeds that change their color turn from green to orange on ripening [14]. Average weight of a seed is 68 mg, and its diameter ranges from 4 to 5 mm [12]. The dragon's blood tree is most famous for the blood-red sap that oozes out of it when cut or injured [15]. Photos of the plant and its resinous material (dragon's blood) are shown in Figure 1.

## 2. Phytochemistry

Phytochemical studies of *D. cinnabari* have led to the isolation of a number of flavonoids [16, 17], biflavonoids [18, 19], a series of sterols and triterpenoids [20], and triflavonoids [21].

### 2.1. Flavonoids

**Figure 2:**
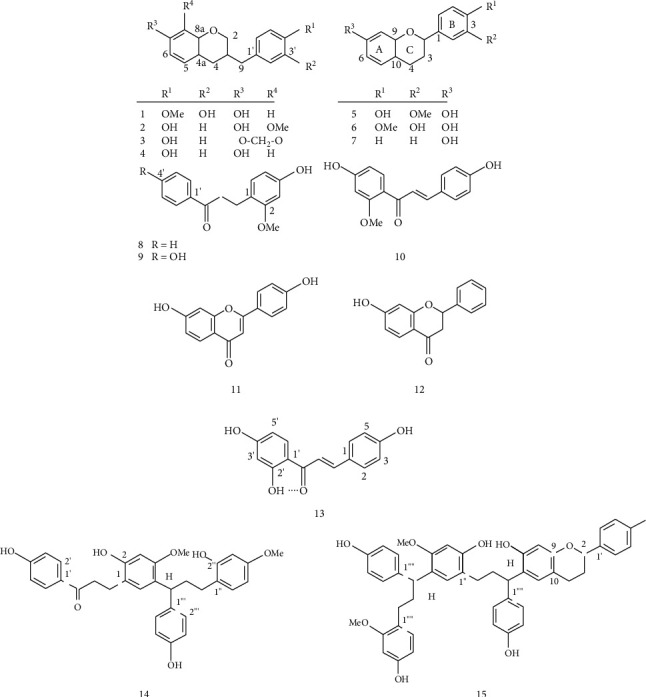
Structures of compounds isolated from *D. cinnabari* resin cleared up by spectroscopic methods.

The resin of *D. cinnabari* contains several flavonoids [16, 17], including 7-hydroxy-3-(3-hydroxy-4-methoxybenzyl)chroman (1); 7-hydroxy-3-(4-hydroxybenzyl)-8-methoxychroman (2); 3-(4-hydroxybenzyl)-8-methyl-enedioxychroman (3); 7-hydroxy-3-(4- hydroxybenzyl)chroman (4); 7,4′-dihydroxy-3′-methoxyflavan (5); 7,3′-dihydroxy-4′-methoxyflavan (6); 7-hydroxyflavan (7); 4-hydroxy-2-methoxydihydrochalcone (8); 4,4′-dihydroxy-2-methoxydihydrochalcone (9); 4,4′-dihydroxy-2′-methoxychalcone (10); 7,4′-dihydroxyflavone (11); and 7-hydroxyflavan-4-one (12). The structures of these compounds have been elucidated by spectroscopic methods and are shown in Figure 2. In addition, the flavonoid 2′,4,4′-trihydroxychalcone (13), determined by Nuclear Magnetic Resonance (NMR) spectroscopy, was isolated as yellow needles for the first time via column chromatography of the *D. cinnabari* fraction [22]. This flavonoid was also isolated from *D. cochinchinensis* and is known as isoliquiritigenin [23, 24]. Sun et al. [25] have reported the presence of 13, 3, and 20 different types of chalcones, chalcanes, and dihydrochalcones, respectively. Among dihydrochalcones, loureirin A and B are considered as indicators for quality control of dragon blood from *D. cinnabari* [26]. Ethyl acetate extract of *D. cinnabari* resin has been identified for the presence of dracidione, a chalcone- dihydrochalcone dimer [27].

### 2.2. Biflavonoids

Previous phytochemical studies of *D. cinnabari* have led to isolation of many bioflavonoids. The biflavonoids 2′-methoxysocotrin-5′-ol, socotrin-4′-ol, and homoisosocotrin-4′-ol were previously isolated from *D. cinnabari* resin, and their structures elucidated mainly by NMR [18]. The recently isolated and identified biflavonoid cinnabarone (14) [19] is composed of a dihydrochalcone and a deoxotetrahydrochalcone moiety connected by a C-C bond. Its structure was determined by NMR and is outlined below.

### 2.3. Triflavonoids and Metacyclophanes

The triflavonoid damalachawin (15) in dragon's blood comprises a flavan and two deoxotetrahydrochalcone moieties [21]. It mainly differs from cinnabarone by replacement of the keto group by a 7-hydroxyflavan-6-yl group and a hydrogen atom. Its structure was identified by NMR as outlined below (Figure 2). In addition, metacyclophanes (dracophane) have also been identified in dragon's blood resin [28].

### 2.4. Sterols and Terpenoids

A total of 13 terpenoids, namely, *α*-Thujene, *α*-Pinene, Camphene, *β*-Pinene, *δ*-3-Carene, p-Cymene, Limonene, (−)-Isodauca-6,9-diene, *γ*-Elemene, trans-Muurola-3,5-diene, *γ*-Humulene, *γ*-Himachelene, and *ω*-Amorphene were reported to be present in resins of *D. cinnabari* [29]. Likewise, Masaoud et al. [20] isolated and identified sterols and triterpenoids present in the resin of *D. cinnabari*. They reported that the resin of this plant contains cholesterol, lupeol, Cholest-4-en-3-one, stigmastanol, betulin, campesterol, 4*α*-methylcholest-7-en-3*β*-ol, 24-methylenecycloartanol, 31-norcycloartanol, stigmast-22-en-3*β*-ol, 4*α*, 14*α*-dimethylcholest-8-en-3*β*-ol, cycloartanol, sitosterol, lanost-7-en-3*β*-ol, and stigmasterol [20].

## 3. Traditional Uses

*D. cinnabari* resin has been traditionally used for a long time in folk medicine as an analgesic, astringent, antiseptic, hemostatic, and antiulcer remedy; to treat diarrhea, fevers, fractures, and burns; and as an abortifacient, if taken during the first trimester of pregnancy [30, 31]. In Socotra, *D. cinnabari* (resin) has also been traditionally used as a therapeutic agent for the treatment of GIT (gastrointestinal tract), skin, eye, and dental diseases [5]. Since centuries, it has been used as a colorant in artworks as found in paints of various ancient reverse glass paintings [15].

## 4. Pharmacological Effects

*D. cinnabari* resin is characterized by the presence of different bioactive flavonoids, which are responsible for its wide pharmacological effects [32]. Al-Afifi et al. [33] reported the tolerability of a methanol extract of *D. cinnabari* resin administered to rats included dosages up to 1500 mg/kg daily for 28 days without any toxic effects.

### 4.1. Hemostasis and Wound Healing

The hemostatic potency of *D. cinnabari* resin is found to be effective against external and internal injuries [5]. There are no scientific studies that demonstrate the exact mechanism by which this resin exerts its wound healing activity; however, wound healing formula containing the resin was patented [34].

Many authors have evaluated the wound healing activity of *Dracaena* species resin using animal models. For example, the ethanolic extract of *D. cochinchinensis* resin showed antithrombotic and anticoagulation activities in rats [31]. Likewise, Liu et al. [35] demonstrated that rats that received an ethanolic extract of *D. cochinchinensis* resin showed significantly better wound contraction and skin-breaking strength compared with the control group. These results highlight the significance of dragon's blood resin in the reduction of clotting time. Other *in vitro* studies have shown an inhibitory effect of three (loureirin B, cochinchinenin A, and 3,4′-dihydroxy-5-methoxystilbene) compounds isolated from dragon's blood on ADP-induced platelet aggregation [36, 37]. In addition, a clinical trial conducted by Namjoyan et al. [38] suggested that dragon's blood resin from *Croton lechleri* is a potent, affordable, and safe healing agent.

### 4.2. Antidiabetic and Hypolipidemic Effects

The resin of *D. cinnabari* has been shown to have high antidiabetic activity through standard glucose uptake procedures against MCF-7 cell lines *in vitro* [39]. Similarly, Al-Baoqai et al. [40] demonstrated that an ethanolic extract of *D. cinnabari* resin (100 and 300 mg/kg) has hypoglycemic and hypolipidemic activity in alloxan-induced diabetic rats. *In vitro* assay of the hypolipidemic effects of another *D. cinnabari* resin extract has inhibition potential against pancreatic lipase, malate dehydrogenase, and glucose-6-phosphate dehydrogenase [41]. Furthermore, a newly C-linked chalcone-dihydrochalcone dimer, named dracidione, isolated from the *D. cinnabari* resin is reported to have moderate *α*-glucosidase inhibitory activity, with a half-maximal inhibitory concentration of 40.27 *μ*g/mL [28]. These results indicate *D. cinnabari* resin has hypoglycemic and antihyperlipidemic effects that can play a role in the treatment of diabetes.

### 4.3. Antimicrobial Effects

Natural products of different higher plants have been reported to be good sources of antimicrobial agents [42]. The first preliminary investigations to show the antimicrobial activity of *D. cinnabari* resin extracts were done by Mothana and Lindequest [43] and Taleb et al. [44]. Also, a dichloromethane extract of *D. cinnabari* resin has been found to have good inhibitory activity against various food-borne pathogens using an agar disc diffusion method [45]. In addition, the antimicrobial activities of different solvent (chloroform, methanol, and benzene) extracts of *D. cinnabari* resin against Gram-positive bacteria, Gram-negative bacteria, and fungi revealed that these microorganisms have varied sensitivity to the different extracts [46, 47]. Similarly, Altwair and Edrah [48] have reported that aqueous and ethanolic extracts of *D. cinnabari* significantly inhibited the activity of *E. coli* (13 & 14 mm), *P. vulgaris* (9 and 10 mm), *P. aeruginosa* (8 and 9 mm), *K. pneumonia* (7 and 8 mm), and *S. saprophyticus* (10 and 11 mm), respectively. Antimicrobial assays have been performed to assess the effect of *D. cinnabari*'s resin extracts against *B. subtilis*, *S. aureus*, *M. luteus*, *S. flexneri*, *S. enteritidis*, *P. mirabilis*, *E. areogenes*, *E. coli*, *P. aeruginosa*, *C. albicans*, *and A. flavus*. Purposely, CH_2_Cl_2_ extract of dragon's blood resin revealed maximum antimicrobial potential against all the tested bacterial and fungal strains except *Salmonella enteritidis* [45]. The methanolic extract of *D. cinnabari* also showed antiviral effects against influenza virus A and herpes simplex virus with IC_50_ values 1.5 *μ*g/mL and 12.5 *μ*g/mL, respectively [49]. The previous documented antimicrobial activity of aqueous and ethanolic extract of *D. cinnabari* may be due primarily to the presence of flavonoids and their antioxidant activity.

### 4.4. Anti-Inflammatory and Analgesic Effects

Alwashli et al. [50] evaluated the anti-inflammatory and analgesic activities of an ethanolic extract of *D. cinnabari* resin using animal models and found that it significantly reduced inflammation at 50 and 150 mg/kg oral doses. In lipopolysaccharide-stimulated mouse macrophage cell line RAW 264.7, methanolic extract of *D. cinnabari* resin and its bioactive component (4′-hydroxy-7,8-methylenedioxyhomoisoflavan) have shown inhibitory effect on nitrite, tumor necrosis factor-*α*, and interleukin-6 production. Reduction in rat edema also validated the anti-inflammatory potential of experimented treatments. These results suggest that *D. cinnabari* resin has important anti-inflammatory effects at selected doses [51]. In addition, compounds isolated and purified from a crude hexane extract of *D. cinnabari* resin were also shown to have anti-inflammatory activity [52].

### 4.5. Enhancing Immune Function

The immune system is a highly complex system with both innate and acquired responses that may be altered by certain biological or pharmacological components; such alteration of immune response is referred as immunomodulating activity [53]. To date, no published research articles have investigated the effect of *D. cinnabari* resin on immune system activity. However, one study conducted on female mice showed that administration of dragon's blood (0.072 g/kg) significantly elevates spleen weight [54]. Furthermore, microscopic examination revealed an enlarged follicular germinal center with a significant increase in plasma, giant, and reticular cell number in the medullary cord of the spleen. These results indicate the importance of dragon's blood in the enhancement of the immune system.

### 4.6. Antispasmodic and Relaxant Effects

The aqueous extract of *D. cinnabari* resin has been shown to cause a concentration-dependent decrease of amplitude in phasic contractions. In albino male rats, it relaxes the tone of longitudinal segments of the ileum, uterus, and urinary bladder rings [30]. This observation is consistent with the reported effects of other *Dracaena* species and dragon's blood-producing plants. The relaxant effect of the *D. cinnabari* resin aqueous extract may be attributed to the presence of hydrophilic flavonoids in the resin since different flavonoids have been shown to exert spasmolytic effects on smooth muscles of different preparations [55]. Following this report, there have been no other publications investigating the relaxation potential of *D. cinnabari* resin extracts except for a registered patent of wound healing formula containing the resin [34]. Meanwhile, there is a report on mice indicating that *D. cochinchinensis* resin antagonizes uterine smooth muscle contraction instigated by diethylstilbestrol [56].

### 4.7. Cardiotonic and Hypotensive Potential

The injection of an aqueous extract of *D. cinnabari* resin (10^−4^–0.03 mg) *in vivo* (rat model) has been shown to increase contractility but did not significantly affect the beating rate of the isolated perfused heart of a rat. Additionally, in anesthetized rats, it revealed a hypotensive effect when intravenously injected (0.04–12 mg/kg) [30].

### 4.8. Anticancer, Antitumor, and Chemopreventive Potential

Various *Dracaena* species have been investigated for anticancer, antitumor, and chemopreventive potential. An *in vitro* microsomal peroxidation assay was used in the first screen of flavonoids and chalcones of *D. cinnabari* resin for antioxidant activity. Among them, 7,8-methylenedioxy-3(4-hydroxybenzyl)chromane was one tested homoisoflavonoid, which exhibited strong antioxidant activity comparable to that of the strongest flavonol antioxidant known, quercetin [57]. In the screening of Yemeni plants used in folk medicine for anticancer potential, methanolic extracts of *Dendrosicyos socotrana*, *Withania aduensis*, *W. riebeckii*, *D. cinnabari*, and *Buxus hildebrandlii* had the greatest anticancer activity against several tumor lines tested [43]. Alabsi et al. [58] designed a bioassay-guided fractionation approach to determine the cytotoxic and apoptosis-inducing effects of *D. cinnabari* resin on human oral squamous cell carcinoma and concluded that it has the potential to be developed as an anticancer agent. *In vitro*, ether and ethyl acetate extracts of *D. cinnabari* resin showed 50% inhibition against MCF-7 breast cancer cells at 100 *μ*g/mL doses [59]. A recent *in vivo* study revealed that administration of a methanol extract of *D. cinnabari* resin at dosages of 100, 500, and 1000 mg/kg in mice decreased the incidence of 4-nitroquinoline-1-oxide-induced oral squamous cell carcinoma compared to the induced cancer group that did not receive treatment [33]. Receipt of 1000 mg/kg extract was shown to inhibit expression of cyclin D1, Ki-67, Bcl-2, and p53 proteins as well as induce apoptosis by the upregulation of *Bax* and *Casp3* and downregulation of *Tp53*, *Bcl-2*, *Cox-2*, *cyclin D1*, and *EGFR* [34]. According to the finding of an i*n vitro* study, the methanolic *D. cinnabari* resin extract induced apoptosis and other cytotoxic effects in H103 tongue squamous cell carcinoma cells in a dose- and time-dependent manner. Methanolic extract of dragon's blood resin has shown a significant cytotoxic effect in H103 cells, while low cytotoxicity was observed in the case of normal cells. In experimented tongue squamous cell carcinoma cells (H103), methanolic extract of *D. cinnabari* resulted in morphological changes, induction of apoptotic conditions, and cell cycle arrest (S and G2/M phase). It was noticed that the incidence of squamous cell carcinoma in induced oral cancer was 85.7%; however, groups that were administrated with methanolic extract (100, 500, and 100 mg kg^−1^) of dragon's blood resin were 57.1%, 28.6%, and 14.3%, respectively [60]. They further stated that this anticancer effect of D. cinnabari may be due to inhibition of p-53, Cox-2 Bcl-2, and cyclin D1 expression and upregulation of Casp-3 and Bax genes [61].

## 5. Conclusions

Here, the botanical source, phytochemistry, flavonoid content, and pharmacological effects of dragon's blood resin from Yemeni *D. cinnabari* plants were reviewed. Information was collected from 61 published articles studying different aspects of dragon's blood resin. The main chemical constituents of *D. cinnabari* resin are flavonoids, which have been demonstrated to have significant blood circulation, hemostasis, muscle relaxation, analgesic, and anticancer effects. Interestingly, *D. cinnabari* resin from Socotra Island has been shown to have more powerful effects than other imported ones available in local Yemeni markets. Studies on various cell lines and animal modeling must be carried out to validate the pharmacological properties of bioactive compounds within the resin. Further investigations regarding volatile metabolites of the resin should also be conducted due to the limited number of current reports on this topic.
